# Diseases of Women

**Published:** 1898-12

**Authors:** 


**Diseases of Women.** By GEORGE ERNEST HERMAN, M.B. Pp. xvi., 886. London: Cassell and Company, Limited. 1898.

We must confess to having mingled feelings with regard to this work. The author has thought it desirable to arrange the maladies according to their leading symptoms—an arrangement confessedly neither logical nor pathological, and one involving some repetition. Perhaps this accounts for the chapters on neurasthenia, hysteria, and headache, which seem to us as much out of place as would be chapters on diabetes or jaundice. It results too in a semi-conversational style, which, pleasant though it may be, lacks the greater detail of a strictly scientific phraseology. Thus, in the operation of vaginal hysterectomy, it is directed that the ligatures should be passed "as far from the uterus as you can," without any specific warning as to the possibility of including the ureters by such a procedure, though the danger of this is referred to incidentally on a subsequent page. Then again, in the chapter on acute general peritonitis, no mention is made of the vermiform appendix, though hernia, perforation of stomach or bowel, &c., are mentioned amongst the "common causes." These are to be regarded as the result of the author's system of classification, for the book as a whole shows a thorough knowledge of the subject resulting from a wide clinical experience. The general principles are sound, and the methods of treatment advocated are well-established and trustworthy.

